# Pharmacologic and surgical therapies for patients with Meniere’s disease: a protocol for a systematic review and meta-analysis

**DOI:** 10.1186/s13643-019-1195-1

**Published:** 2019-12-30

**Authors:** Nadera Ahmadzai, Wei Cheng, Dianna Wolfe, Jamie Bonaparte, David Schramm, Elizabeth Fitzpatrick, Vincent Lin, Becky Skidmore, Leila Esmaeilisaraji, Shaun Kilty, Brian Hutton

**Affiliations:** 1 Center for Practice Changing Research, Ottawa Hospital Research Institute, 501 Smyth Road, Box 201B, Ottawa, ON K1H 8 L6 Canada; 2 Department of Otolaryngology-Head and Neck Surgery, The Ottawa Hospital, 501 Smyth Rd, Ottawa, ON K1H 8L6 Canada; 3 Faculty of Health Sciences, University of Ottawa, Ottawa, Canada; 4 Children’s Hospital of Eastern Ontario Research Institute, Ottawa, Canada; 5 Department of Otolaryngology – Head & Neck Surgery, Sunnybrook Research Institute, Sunnybrook Health Sciences Centre, Toronto, Canada; 6 Faculty of Medicine, University of Toronto, Toronto, Canada; 7 Medicine Prof. Corp, Ottawa, Canada; 8 The University of Ottawa Faculty of Epidemiology and Community Medicine, 451 Smyth Rd, Ottawa, ON K1H 8L1 Canada

**Keywords:** Meniere’s disease, Meta-analysis, Network meta-analysis, Systematic review

## Abstract

**Background:**

Hearing loss is one of the leading causes of disability in Canada and worldwide, with more than one million Canadians enduring a hearing-related disability. Meniere’s disease (MD) is a chronic condition of the inner ear, manifesting as a triad of disabling symptoms, including attacks of vertigo, fluctuating sensorineural hearing loss (SNHL), and tinnitus. Impacts on quality of life are severe, particularly with respect to restrictions in social participation and physical activity, fatigue, and reduced capacity to work. Anxiety and other psychological disorders may result from the restrictions imposed on life, the constant uncertainty of vertigo attacks, and fluctuating SNHL, with neuroses and depression affecting 40 to 60% of sufferers of intractable MD. There is a need to establish the benefits of previously studied interventions with greater certainty. The planned systematic review and meta-analyses/network meta-analyses (NMAs) will assess the relative effects of competing pharmacologic and surgical interventions for management of MD in adults.

**Methods:**

An experienced medical information specialist in consultation with the review team will develop the electronic search strategies. We will search various databases including MEDLINE, Embase, and the Cochrane Library with no date or language restrictions for published literature, and key clinical trial registries for in-progress and completed trials. Screening of the literature will be performed by two reviewers independently using pre-specified eligibility criteria, and quality of the included studies will be assessed using the Cochrane Risk of Bias tool. We will resolve disagreements through consensus or third-party adjudication. When applicable, meta-analyses and NMAs will be pursued to compare interventions in terms of their effects on outcomes, including frequency and severity of vertigo, occurrence and intensity of tinnitus, changes in hearing and speech recognition, quality of life, and harms. Separate analyses exploring the effects of pharmacologic and surgical approaches will be performed.

**Discussion:**

Our planned systematic review will provide informative evaluations of existing treatments for management of Meniere’s disease. The findings will inform practitioners as to the relative benefits and harms of the existing competing interventions for MD, offer optimal clinical treatment strategies, identify evidence gaps, and determine promising therapies for evaluation in future trials.

**Systematic review registration:**

PROSPERO CRD42019119129

## Background

Hearing loss is one of the leading causes of disability in Canada and worldwide, with more than one million Canadians enduring a hearing-related disability [1, 2]. Despite the availability of various interventions, Canadians with hearing loss may endure a diminished quality of life [3]. Meniere’s disease (MD) is a condition that frequently causes hearing loss and may have a large emotional and financial toll on patients, their families, and society that is often underestimated [4]. MD has a variable clinical course [5]. For instance, hearing loss may spontaneously increase with a concurrent increase in the associated symptomatology of the disease, including potentially incapacitating vertigo. The spontaneous changes in the symptomatology of MD, including further hearing loss, make treatment particularly difficult when the disease symptoms are continually changing and the changes are not predictable.

MD is a chronic condition of the inner ear, manifesting as a triad of disabling symptoms, including attacks of vertigo, fluctuating sensorineural hearing loss (SNHL), and tinnitus [6]. Impacts on quality of life can be severe, particularly with respect to restrictions in social participation and physical activity, fatigue, and reduced capacity to work. Anxiety and other psychological disorders may result from these restrictions on life, the constant uncertainty of vertigo attacks, and fluctuating SNHL [6, 7], with neuroses and depression affecting 40 to 60% of sufferers of intractable MD [8]. The symptoms of MD result from an increase in the hydraulic pressure within the inner ear endolymphatic system, termed “endolymphatic hydrops” (EH). Currently, MD diagnosis is symptom based, with guidelines that define diagnostic criteria and certainty [9]. Management of MD focuses on treatment and prevention of vertigo attacks, improvement or preservation of hearing and vestibular function, and prevention of bilateral MD [10]. Both medical and surgical interventions have been used [11]. Pharmaceutical interventions include systemic (e.g., diuretics, antihistamines) as well as intra-tympanic therapies (e.g., gentamicin, steroids) aimed at either reducing pressure in the endolymphatic system or chemical labyrinthectomy [10, 11]. Dietary therapies to reduce endolymphatic pressure include restrictions of salt, water, alcohol, or caffeine [10]. Surgical treatment involves conservative procedures in which the hearing preservation is attempted or destructive labyrinthectomy in which hearing is lost [10]. Psychological support may improve results of treatment for patients with intractable MD [8].

The detrimental effects of MD on patients’ quality of life may be severe, and thus early detection and rapid intervention are encouraged [7]. However, the uncertainty surrounding the efficacy of various available interventions makes the selection of appropriate treatments difficult. This protocol presents methodology for a systematic review incorporating meta-analyses/network meta-analyses (NMAs) that was developed by researchers in collaboration with clinical content experts who prioritized the question of interest.

## Objectives

The primary objective of this systematic review is to assess the relative effects of available pharmacologic therapies in patients with MD on vertigo and other key patient outcomes in randomized controlled trials and quasi-randomized trials. The secondary objective is to assess the effects of surgical interventions in the MD population on vertigo and other key patient outcomes in randomized and quasi-randomized trials.

## Methods

This protocol adheres to the standards of the Preferred Reporting Items for Systematic Review and Meta-Analysis Protocols (PRISMA-P) Statement [12], and is registered with the International Prospective Register of Systematic Reviews (PROSPERO) database (CRD42019119129) [13]. A populated PRISMA-P checklist for this protocol is provided as an additional file (see Additional file 1). Any post hoc modifications to the plans presented within the protocol will be recorded and described in the publication of the final report to ensure transparency. The final report will be developed in consultation with the PRISMA Extension Statement for NMA to ensure all aspects of methods and findings are fully reported [14].

### Study eligibility criteria

We have established the review eligibility criteria based on the PICOS (Population-Intervention-Comparators-Outcomes-Study design) framework. We will include primary studies that meet the following criteria:

- Population. Studies enrolling adult patients with MD per established criteria (i.e., American Academy of Otolaryngology-Head and Neck Surgery (AAO-HNS) [9]) receiving pharmacologic or surgical interventions for their condition (e.g., endolymphatic sac decompression, intra-tympanic gentamicin injection, or others as detailed below) will be sought [15]. In studies with enrollment criteria that required patients to be unresponsive to a prior intervention in order to be treated with the intervention(s) of interest, we will discuss the potential for important clinical heterogeneity with our clinical experts and exercise discretion about whether these studies can be appropriately combined together in quantitative analyses.
- Interventions/comparators***.*** The following interventions will be of interest:
  - ○ Systemic pharmaceuticals: diuretics (e.g., hydrochlorothiazide, furosemide), Motion sickness/anti-nausea medications (e.g., anticholinergics, antihistamines (betahistine), phenothiazines), benzodiazepines (e.g., diazepam);
  - ○ Intra-tympanic pharmaceuticals: intra-tympanic gentamicin, intra-tympanic steroids;
  - ○ Surgical interventions: sacculotomy, vestibular nerve section, labyrinthectomy, tympanostomy tube, endolymphatic duct blockage, endolymphatic sac decompression, endolymphatic shunt, transtympanic pressure treatment.

While we have structured interventions into broad categories (e.g., systemic and intra-tympanic pharmaceuticals, surgical, and others), our primary analyses will consider interventions at a more granular level to ensure findings are of maximal clinical relevance. We will pursue separate sets of analyses relating to pharmacologic and surgical interventions, as this approach is in alignment with clinical decision-making. After study data has been extracted, we will consult our clinical experts to determine whether additional treatment nodes or other modifications related to network geometry are needed to enhance representativeness of interventions (e.g., considering variable doses/durations of pharmaceutical therapy). If combination therapies are encountered, we will include them as additional treatment groups in the analyses to be performed.

- Outcomes. Endpoints of interest will include the following:
  - ○ frequency, severity, type, and control of vertigo measured via electrocochleography score test or other methods;
  - ○ occurrence and intensity of tinnitus measured via various methods such as psychoacoustic tests (pitch match, loudness match, maskability, residual inhibition, etc.), rating scales (e.g., verbal rating scale, numerical rating scale, visual analog scale, poster style, and mechanical device), questionnaires describing functional effects (e.g., tinnitus questionnaire, tinnitus handicap questionnaire, tinnitus severity scale, subjective tinnitus severity scale/tinnitus reaction questionnaire, tinnitus severity grading, tinnitus severity index, tinnitus handicap inventory, intake interview for tinnitus retraining therapy), and patients’ global perception of treatment-related changes;
  - ○ changes in hearing, based on Pure Tone Average (PTA) in decibels, and speech recognition, such as word recognition score (WRS) that may also be labeled as speech discrimination score (SDS), and speech reception threshold (SRT);
  - ○ quality of life measured by various scales such as Quality of Well-being Scale (QWB), SF-12, Physical SF-12 score; Mental SF-12 score, Center for Epidemiologic Studies–Depression Scale (CESD);
  - ○ perception of aural fullness;
  - ○ and harms, including hearing loss, withdrawals due to adverse effects, and serious side effects defined by authors.
- Study design**.** Randomized controlled trials and quasi-randomized trials
- Timing. Studies with a minimum follow-up duration of 6 months after the first intervention
- Language. English language
- Setting. Any setting

### Information sources and search strategy

The search strategies will be developed and tested through an iterative process by an experienced medical information specialist in consultation with the review team. The strategies will be peer reviewed by another senior information specialist prior to execution using the PRESS Checklist [16] Using the OVID platform, we will search Ovid MEDLINE®, including Epub Ahead of Print and In-Process & Other Non-Indexed Citations, and Embase Classic+Embase. We will also search the Cochrane Library on Wiley.

Strategies will utilize a combination of controlled vocabulary (e.g., “Meniere Disease,” “Endolymphatic Hydrops”) and keywords (e.g., “auditory vertigo,” “endolymphatic hydrops,” “Meniere’s”). Results will be filtered using headings for systematic reviews, randomized controlled trials, and non-randomized controlled trials as applicable for each database. Vocabulary and syntax will be adjusted across databases. There will be no language or date restrictions on any of the searches, but when possible, animal-only and opinion pieces will be removed from the results. Potentially relevant articles published in languages other than English will be included in an Additional file. We will document any unforeseen limitations pertaining to search strategy upon its completion.

A gray literature search of targeted clinical trial registries, ClinicalTrials.gov, and the International Clinical Trials Registry Platform will also be undertaken. Studies included in existing reviews will be inspected to confirm no relevant studies have been missed. Content experts will be contacted to obtain information on unknown or ongoing studies. The proposed database search strategies are provided in Additional file 3.

### Screening and data extraction

Screening will be performed in two stages via two reviewers working independently and in duplicate against eligibility criteria established a priori, using an online systematic review software program (Distiller Systematic Review (DSR) Software; Evidence Partners Inc, Ottawa, Canada). Stage 1 screening will be based on review of the abstracts and titles identified from the electronic search, while stage 2 screening will consider full-text review of the articles deemed potentially relevant during stage 1. Screening at both stages will commence with a calibration exercise to ensure consistent application of eligibility criteria. A screening pilot will be performed prior to full screening of titles and abstracts (25 titles and abstracts) and full-text screening (25 studies). At stage 1, two reviewers (NA and LE) will independently assess the titles and abstracts for eligible studies using the liberal accelerated method [17] where only one reviewer is required to include citations for further assessment at full-text screening and two reviewers are needed to exclude a citation. At stage 2, the full-text articles of potentially relevant citations will be retrieved for full-text screening and the same two reviewers (NA and LE) will independently assess the article for relevancy. Disagreements between reviewers will be resolved via consensus or third-party adjudication. The study selection process will be reported using a PRISMA flow diagram [18] in the final publication. References of all included studies will be scanned for inclusion, by one reviewer. At least one content expert will be consulted for additional studies. Study authors will be consulted where necessary for verifying eligibility and for missing or unclear information on studies (and information will be included if received in a timely manner). A list of the excluded studies alongside the rationale for their exclusion will be provided in an additional file to the completed review. With regard to duplicate publications, companion documents, or multiple reports of a primary study, we will collate all available data and use the most complete set and exclude the duplicate version or companion documents with no additional data.

A standardized data extraction form in Microsoft Excel (Microsoft Corporation, Seattle, WA, USA) will be used for collecting key study information that includes all pre-specified data items (see Additional file 2). After piloting the data extraction form on a small number of studies, two reviewers (NA and LE) will extract the data independently and any discrepancies will be resolved by discussion or a third person. Information from each study will be recorded that will include (but not be limited to) the following: publication characteristics (e.g., authors’ names, publication year, and journal), study design traits (cited trial design, clinical setting, duration of follow-up, number of patients randomized and number analyzed for each outcome, occurrence of dropouts, funding source, and authors’ conflict of interest etc.), study population details (patient inclusion and exclusion criteria, age, sex, body mass index (BMI), race, comorbidities, prior treatments, and relevant baseline data, such as initial PTA, WRS/SDS, hearing loss in the opposite ear previously affected with MD, aural fullness in the opposite ear, time from onset to treatment, prior otologic surgery, presence of tinnitus and vertigo, etc.), intervention and comparator specifics (type, dose, unit, duration, frequency, route of administration, strategy of administration, and co-intervention, etc.), and outcome data (including reported outcome definitions and summary data related to treatment effects (e.g., mean change and the corresponding standard error for continuous outcomes, and number of events and number of total patients for dichotomous outcomes), and reported scales for evaluating the outcomes). A complete list of pre-specified data items is presented in Additional file 2. Means and measures of dispersion will be approximated from figures in the primary studies using online tools. When available, data from both intention to treat and per protocol analyses will be extracted. We will contact authors for any missing or additional data of interest. Authors’ defined pre-specified outcomes of interest will be extracted and grouped accordingly for analyses.

### Outcomes and prioritization

We explored the Core Outcome Measures in Effectiveness Trials (COMET) initiative [19] but did not locate a core outcome set for Meniere’s disease [20]. As such, the endpoints of interest for this review were selected via consultation with our clinical experts. The primary outcome of interest will be vertigo, while the secondary outcomes of interest will include changes in hearing, tinnitus, quality of life, aural fullness, and harms. With regard to outcomes definitions, we will gather outcomes with any definitions provided by the primary study authors. We will group together the data with similar definitions across the studies. Priority will be given to established definitions where possible. For instance, for vertigo control, we will consider the definition from the American Academy of Otolaryngology-Head and Neck Surgery (AAO-HNS) [9]. However, in the event of insufficient outcome data using this definition, we will consider other definitions in consultation with our clinical context experts, and the set with the most available data and clinical relevance will be given priority in analysis. In terms of time of assessment for the endpoints of interest, we will consider data reported according to the AAO-HNS recommendation on reporting the treatment results [9] when available. For instance, for frequency of definitive attacks, outcome data from the period 6 months before treatment should be compared with interval occurring between 18 and 24 months post-treatment. However, if insufficient data are reported based on AAO-HNS criteria across primary studies, we will collect outcome data at various time points such as baseline, 6 months, 12 months, and 24 months post-treatment if possible. In the absence of sufficient data, we may consider other reported time points in consultation with our clinical content experts to ensure clinical relevance.

### Risk of bias assessment

We will use the Cochrane Risk of Bias Tool for RCTs [21] to evaluate the risk of bias of each included RCT and quasi-randomized trial. Two reviewers will carry out assessments independently and resolve disagreements via consensus or third-party adjudication. All domains of the Cochrane Risk of Bias tool for RCTs will be considered, including selection bias (sequence generation and allocation sequence concealment), performance bias (blinding of participants and personnel), detection bias (blinding of outcome assessment), attrition bias (incomplete outcome data), reporting bias (selective reporting), and other biases considered relevant to the review topic. We will also evaluate baseline imbalances between groups with respect to comorbidities and factors that may impact our outcomes of interest, including history of falls more than once in the past year, older age, white race, female sex, higher BMI, current tinnitus, prior therapies received, allergies, immune dysfunction (e.g., ankylosing spondylitis, systemic lupus erythematosus, psoriasis), autonomic dysfunction, poor mental health [18, 20, 22], arthritis [23], history of hearing loss or episodic vertigo [24], familial history of MD, and diabetes mellitus [25].

### Approaches to evidence syntheses

A. Criteria for quantitative synthesis

To assess the assumptions for conducting NMA (i.e., the transitivity assumption, relating to similarity amongst studies in an evidence network), a variety of information related to study methods, patient demographics, and eligibility criteria will be collected. These will include the following: mean age at onset, percent female patients, mean disease duration, frequency/severity measures of vertigo at baseline, percent with history of migraine headaches, percent with previously identified tinnitus, measures of average tinnitus intensity, and other factors. These study features will be reviewed with our clinical experts using a combination of table summaries, box plots, and bar plots to identify potential outlier studies that may warrant exclusion from formal analyses.

As noted earlier, separate sets of analyses pertaining to comparison of pharmacologic interventions and surgical interventions will be performed. Initially, we will inspect the characteristics of included studies such as patients’ clinical characteristics (age, sex, and clinical history, including duration of hearing impairment and baseline severity) and methodologic homogeneity (e.g., risk of bias, study design), and we will summarize them accordingly. A pairwise meta-analysis for each intervention comparison will be pursued to explore statistical heterogeneity (based upon the *I*^2^ statistic) if data permit.

B. Planned quantitative analysis

If data permits, both fixed effects and random effects Bayesian NMAs will be performed to compare interventions contained within the included studies that are sufficiently connected for a specified clinical endpoint. A common between-trial standard deviation will be used for Bayesian NMAs as per established methods [26–28]. Model fit will be assessed by comparing total residual deviance with the number of unconstrained data points [29] and will be considered adequate if these quantities are approximately equal. The deviance information criteria (DIC) will be used for selection between models, with a difference of five points suggesting an important difference [29] (with smaller values being preferred). The type of endpoint under analysis (e.g., continuous or binary) will determine the use of specific NMA models. We will use mean change per arm (between pre- and post-intervention) for the analysis of continuous endpoints measured in the same units, and the corresponding effect size will be the mean difference. It is common in general that studies report findings of the same continuous endpoint in different formats, some reporting mean changes with corresponding standard errors (SEs), while others report only mean values at baseline and post-treatment with corresponding standard deviations (SDs) for each treatment arm. For the latter scenario, we will consider the appropriateness of assuming a correlation between mean values at baseline and follow-up and calculate the mean changes and corresponding SEs when they are not reported. If we encounter continuous endpoints that are measured using different scales across studies (e.g., a visual analog scale from 0 to 100 versus an itemized, composite score scale to assess severity of vertigo attacks), a model for estimating the effect size as a standardized mean difference (SMD) will be considered to explore benefits across related scales and maximize usage of available data. Estimates of effect sizes for binary endpoints will be expressed as odds ratios. All pairwise comparisons between interventions will be expressed with 95% credible intervals. Key secondary measures of effect, such as the surface under the cumulative ranking curve (SUCRA) and average treatment rankings [30], will be estimated to explore potential orderings of treatments. All NMAs will be carried out using OpenBUGS version 3.2.3 [31] and the R2OpenBUGS package [32] version 3.2-3.2 in R [31].

We will rely on pairwise meta-analyses if the evidence networks of pharmacologic interventions or surgical interventions are not well-connected. Whenever data permit for each pairwise comparison, we will use funnel plots to assess for small-study effects as a signal of publication bias. Where protocols are available, they will be reviewed to inform evaluations for selective reporting within the set of included studies.

C. Proposed additional analyses

If feasible (based upon quantity of available evidence and rigor or reporting of the included studies), we will explore subgroup analyses [27, 33] to evaluate robustness of our findings and the impact of covariates. In consultation with our clinical experts, the subgroups will be chosen and may include (but not be limited to) gender distribution (e.g., percent females), age (older versus younger), BMI (higher BMI versus lower BMI), race (white versus others), presence of dizziness, number of days since initial treatment (or onset of MD), severity of initial hearing loss, type of MD (unilateral versus bilateral), and types of unilateral MD [34]: (1) classic MD (sporadic MD, without migraine and autoimmune disorder), (2) delayed MD (hearing loss antedates vertigo episodes for months or years and is without migraine or autoimmune disorder in most cases), (3) familial MD, (4) MD with presence of migraine, and (5) MD comorbid with autoimmune disorder.

D. Narrative summary

If excessive heterogeneity is identified and the research team feels meta-analysis is inappropriate, a narrative summary of findings with supporting tables and figures will be prepared.

## Discussion

To date, there have been a variety of pharmaceuticals used for the treatment of MD, including systemic drugs and intra-tympanic medications as well as surgical approaches. There exist systematic reviews comparing effectiveness of pairs of interventions in patients with MD [35–38]. However, these systematic reviews focus only on direct pairwise comparisons (e.g., one intervention versus placebo or another active treatment) [39–41]. When applicable, NMA compares multiple interventions in one analysis and borrows strength from both direct and indirect evidence. Our planned systematic review will employ sound methodology and will provide new and informative evaluations of present competing therapies and their relative benefits in managing MD. As interventions for MD are often chosen using a staged approach, we have planned to focus our review upon separate comparisons of two important aspects of treatment which typically follow insufficient response to lifestyle modifications: pharmacologic interventions and surgical interventions. An important strength of this systematic review (if sufficient data and homogeneity are present) will be the performance of NMA of therapies for the management of MD; to our awareness, this will represent the first such analysis. A second strength will be the assessment of benefits and harms of not only surgical interventions, but also pharmaceutical agents in treating this condition. A potential challenge will be threats to clinical homogeneity across studies including variability in prior treatments received and other relevant factors.

We will publish the findings of our review in a clinical specialty journal to enhance outreach to clinicians pursuing prospective research on MD. We will report evidence networks collating completed studies as well as ongoing trials identified from www.clinicaltrials.gov and other registries to establish the current state of the evidence. In keeping with the COMET initiative’s recommendation regarding the establishment of standards for outcome selection [42], this review will also appraise the inclusiveness of endpoint measurement over time. In addition to publications, we also plan to develop lay summaries that will be disseminated online and distributed to patient groups and key societies.

## Supplementary information

**Additional file 1.** PRISMA checklist.

**Additional file 2.** Search strategy.

**Additional file 3.** Data extraction form.

## Data Availability

Not applicable

## Abbreviations

MD: Meniere disease
NMA: Network meta-analysis
PICOs: Population, intervention, comparator, outcome, study design
PRESS: Peer review of electronic search strategies
PTA: Pure tone average
SDS: Speech discrimination score
SMD: Standardized mean difference
SR: Systematic review
SRT: Speech reception threshold
WRS: Word recognition score
