# Supplementary material for: Pharmacologic and surgical therapies for patients with Meniere’s disease: a protocol for a systematic review and meta-analysis
Source: Syst Rev. 2019 Dec 30;8:341. doi: 10.1186/s13643-019-1195-1 (PMC6937806; doi:10.1186/s13643-019-1195-1)
Supplement: Supplementary file 3 — Additional file 3. Data extraction form. [file 13643_2019_1195_MOESM3_ESM.docx]

# Additional file 3: Search Strategy

*RCTs*

Ovid Multifile

Database: Embase Classic+Embase

--------------------------------------------------------------------------------

1 Endolymphatic Hydrops/ (9377)

2 Meniere Disease/ (17205)

3 meniere*.tw,kf. (13834)

4 ((acoustic* or auditory or aural or otogenic*) adj3 vertigo*).tw,kf. (461)

5 ((cochlea* or endolymphatic* or labyrinth*) adj3 hydrop?).tw,kf. (3547)

6 labyrinth* syndrome?.tw,kf. (83)

7 (labyrinth* adj3 vertigo*).tw,kf. (335)

8 or/1-7 [Meniere Disease] (20688)

9 (controlled clinical trial or randomized controlled trial or pragmatic clinical trial).pt. (612342)

10 clinical trials as topic.sh. (202533)

11 exp Randomized Controlled Trials as Topic/ (274514)

12 (randomi#ed or randomly or RCT? or placebo*).tw,kf. (2075293)

13 ((singl* or doubl* or trebl* or tripl*) adj (mask* or blind* or dumm*)).tw,kf. (388015)

14 trial.ti. (451544)

15 or/9-14 (2698677)

16 8 and 15 [MENIERE DISEASE - RCTS] (722)

17 exp Animals/ not (exp Animals/ and Humans/) (15861665)

18 16 not 17 [ANIMAL-ONLY REMOVED] (600)

19 (comment or editorial or interview or news or newspaper article).pt. (1873915)

20 (letter not (letter and randomized controlled trial)).pt. (2056405)

21 18 not (19 or 20) [OPINION PIECES REMOVED] (595)

22 21 use medall [MEDLINE RECORDS] (339)

23 inner ear disease/ (10429)

24 meniere disease/ (17205)

25 meniere*.tw,kw. (14090)

26 ((acoustic* or auditory or aural or otogenic*) adj3 vertigo*).tw,kw. (435)

27 ((cochlea* or endolymphatic* or labyrinth*) adj3 hydrop?).tw,kw. (3645)

28 labyrinth* syndrome?.tw,kw. (83)

29 (labyrinth* adj3 vertigo*).tw,kw. (410)

30 or/23-29 [Meniere Disease] (29712)

31 randomized controlled trial/ or controlled clinical trial/ (1280118)

32 exp "clinical trial (topic)"/ (265288)

33 (randomi#ed or randomly or RCT? or placebo*).tw,kw. (2077238)

34 ((singl* or doubl* or trebl* or tripl*) adj (mask* or blind* or dumm*)).tw,kw. (388160)

35 trial.ti. (451544)

36 or/31-35 (2844524)

37 30 and 36 [MENIERE DISEASE - RCTS] (1127)

38 exp animal experimentation/ or exp animal model/ or exp animal experiment/ or nonhuman/ or exp vertebrate/ (48371189)

39 exp human/ or exp human experimentation/ or exp human experiment/ (37923009)

40 38 not 39 (10449902)

41 37 not 40 [ANIMAL-ONLY REMOVED] (1070)

42 editorial.pt. (1037613)

43 letter.pt. not (randomized controlled trial/ and letter.pt.) (2051615)

44 41 not (42 or 43) [OPINION PIECES REMOVED] (1058)

45 44 use emczd [EMBASE RECORDS] (689)

46 22 or 45 [BOTH DATABASES] (1028)

47 remove duplicates from 46 (760)

48 47 use medall [MEDLINE UNIQUE RECORDS] (298)

49 47 use emczd [EMBASE UNIQUE RECORDS] (462)

***************************

Reviews

Ovid Multifile

Search Strategy:

--------------------------------------------------------------------------------

1 Endolymphatic Hydrops/ (9377)

2 Meniere Disease/ (17205)

3 meniere*.tw,kf. (13834)

4 ((acoustic* or auditory or aural or otogenic*) adj3 vertigo*).tw,kf. (461)

5 ((cochlea* or endolymphatic* or labyrinth*) adj3 hydrop?).tw,kf. (3547)

6 labyrinth* syndrome?.tw,kf. (83)

7 (labyrinth* adj3 vertigo*).tw,kf. (335)

8 or/1-7 [Meniere Disease] (20688)

9 meta analysis.pt. (98200)

10 exp meta-analysis as topic/ (56671)

11 (meta-analy* or metanaly* or metaanaly* or met analy* or integrative research or integrative review* or integrative overview* or research integration or research overview* or collaborative review*).tw,kf. (308949)

12 (systematic review* or systematic overview* or evidence-based review* or evidence-based overview* or (evidence adj3 (review* or overview*)) or meta-review* or meta-overview* or meta-synthes* or "review of reviews" or technology assessment* or HTA or HTAs).tw,kf. (361956)

13 exp Technology assessment, biomedical/ (23592)

14 (cochrane or health technology assessment or evidence report).jw. (35766)

15 (network adj (MA or MAs)).tw,kf. (16)

16 (NMA or NMAs).tw,kf. (3976)

17 indirect* compar*.tw,kf. (4369)

18 (indirect treatment* adj1 compar*).tw,kf. (562)

19 (mixed treatment* adj1 compar*).tw,kf. (1268)

20 (multiple treatment* adj1 compar*).tw,kf. (300)

21 (multi-treatment* adj1 compar*).tw,kf. (3)

22 simultaneous* compar*.tw,kf. (2159)

23 mixed comparison?.tw,kf. (42)

24 or/9-23 (648468)

25 8 and 24 [MENIERE DISEASE - REVIEWS] (200)

26 exp Animals/ not (exp Animals/ and Humans/) (15861665)

27 25 not 26 [ANIMAL-ONLY REMOVED] (152)

28 (comment or editorial or interview or news or newspaper article).pt. (1873915)

29 (letter not (letter and randomized controlled trial)).pt. (2056405)

30 27 not (28 or 29) [OPINION PIECES REMOVED] (145)

31 30 use medall [MEDLINE RECORDS] (87)

32 inner ear disease/ (10429)

33 meniere disease/ (17205)

34 meniere*.tw,kw. (14090)

35 ((acoustic* or auditory or aural or otogenic*) adj3 vertigo*).tw,kw. (435)

36 ((cochlea* or endolymphatic* or labyrinth*) adj3 hydrop?).tw,kw. (3645)

37 labyrinth* syndrome?.tw,kw. (83)

38 (labyrinth* adj3 vertigo*).tw,kw. (410)

39 or/32-38 [Meniere Disease] (29712)

40 meta-analysis/ (238596)

41 "systematic review"/ (161100)

42 "meta analysis (topic)"/ (38390)

43 (meta-analy* or metanaly* or metaanaly* or met analy* or integrative research or integrative review* or integrative overview* or research integration or research overview* or collaborative review*).tw,kw. (311608)

44 (systematic review* or systematic overview* or evidence-based review* or evidence-based overview* or (evidence adj3 (review* or overview*)) or meta-review* or meta-overview* or meta-synthes* or "review of reviews" or technology assessment* or HTA or HTAs).tw,kw. (364853)

45 biomedical technology assessment/ (22449)

46 (cochrane or health technology assessment or evidence report).jw. (35766)

47 (network adj (MA or MAs)).tw,kw. (16)

48 (NMA or NMAs).tw,kw. (3995)

49 indirect* compar*.tw,kw. (4424)

50 (indirect treatment* adj1 compar*).tw,kw. (565)

51 (mixed treatment* adj1 compar*).tw,kw. (1280)

52 (multiple treatment* adj1 compar*).tw,kw. (302)

53 (multi-treatment* adj1 compar*).tw,kw. (3)

54 simultaneous* compar*.tw,kw. (2159)

55 mixed comparison?.tw,kw. (43)

56 or/40-55 (700823)

57 39 and 56 [MENIERE DISEASE - REVIEWS] (299)

58 exp animal experimentation/ or exp animal model/ or exp animal experiment/ or nonhuman/ or exp vertebrate/ (48371189)

59 exp human/ or exp human experimentation/ or exp human experiment/ (37923009)

60 58 not 59 (10449902)

61 57 not 60 [ANIMAL-ONLY REMOVED] (298)

62 editorial.pt. (1037613)

63 letter.pt. not (randomized controlled trial/ and letter.pt.) (2051615)

64 61 not (62 or 63) [OPINION PIECES REMOVED] (288)

65 64 use emczd [EMBASE RECORDS] (184)

66 31 or 65 [BOTH DATABASES] (271)

67 remove duplicates from 66 (187)

68 67 use medall [MEDLINE UNIQUE RECORDS] (70)

69 67 use emczd [EMBASE UNIQUE RECORDS] (117)

***************************

Cochrane Library

ID Search Hits

#1 [mh ^"Endolymphatic Hydrops"] 7

#2 [mh "Meniere Disease"] 148

#3 meniere*:ti,ab,kw 342

#4 ((acoustic* or auditory or aural or otogenic*) near/3 vertigo*):ti,ab,kw 15

#5 ((cochlea* or endolymphatic* or labyrinth*) near/3 hydrop*?):ti,ab,kw 26

#6 (labyrinth* next syndrome?):ti,ab,kw 0

#7 (labyrinth* near/3 vertigo*):ti,ab,kw 5

#8 {or #1-#7} 357

DSR - 8 [Reviews]

DARE – 7 [Reviews]

CENTRAL – 333 [RCTs]

HTA – 6 [Reviews]
